# Application of Nanoparticles and Nanomaterials in Thermal Ablation Therapy of Cancer

**DOI:** 10.3390/nano9091195

**Published:** 2019-08-24

**Authors:** Zhannat Ashikbayeva, Daniele Tosi, Damir Balmassov, Emiliano Schena, Paola Saccomandi, Vassilis Inglezakis

**Affiliations:** 1 Environmental Science & Technology Group (ESTg), Chemical & Materials Engineering Department, Nazarbayev University, 53 Kabanbay batyr ave., 010000 Nur-Sultan, Kazakhstan; 2 PI National Laboratory Astana, Nazarbayev University, 53 Kabanbay batyr ave., 010000 Nur-Sultan, Kazakhstan; 3 Department of Pedagogical Sciences, Astana International University, 8 Kabanbay batyr ave., 010000 Nur-Sultan, Kazakhstan; 4 Measurements and Biomedical Instrumentation Lab, Department of Engineering, Università Campus Bio-Medico di Roma, Via Alvaro del Portillo, 21-00128 Roma, Italy; 5 Department of Mechanical Engineering, Politecnico di Milano, Via Giuseppe La Masa 1, 20156 Milano, Italy; 6 The Environment & Resource Efficiency Cluster (EREC), Nazarbayev University, 53 Kabanbay batyr ave., 010000 Nur-Sultan, Kazakhstan

**Keywords:** magnetic nanoparticles, gold nanoparticles, nanocomposites, nanorods, nanoshells, carbon nanotubes, cancer tumor, thermal ablation, photothermal ablation, laser ablation

## Abstract

Cancer is one of the major health issues with increasing incidence worldwide. In spite of the existing conventional cancer treatment techniques, the cases of cancer diagnosis and death rates are rising year by year. Thus, new approaches are required to advance the traditional ways of cancer therapy. Currently, nanomedicine, employing nanoparticles and nanocomposites, offers great promise and new opportunities to increase the efficacy of cancer treatment in combination with thermal therapy. Nanomaterials can generate and specifically enhance the heating capacity at the tumor region due to optical and magnetic properties. The mentioned unique properties of nanomaterials allow inducing the heat and destroying the cancerous cells. This paper provides an overview of the utilization of nanoparticles and nanomaterials such as magnetic iron oxide nanoparticles, nanorods, nanoshells, nanocomposites, carbon nanotubes, and other nanoparticles in the thermal ablation of tumors, demonstrating their advantages over the conventional heating methods.

## 1. Introduction

**Figure 1:**
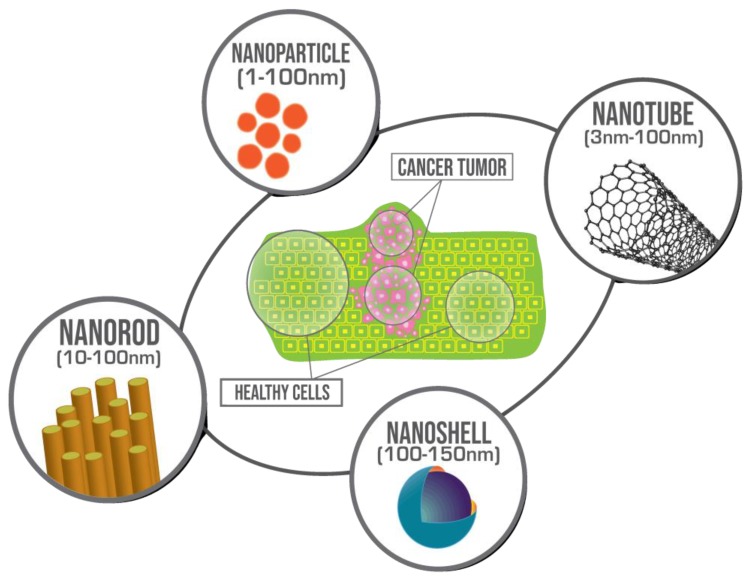
Representation of different types of nanomaterials varied by a shape that can be used for biomedical applications and cancer tumor treatment.

Nowadays, cancer is the most serious health issue leading to the high rates of death worldwide. According to the statistics from the World Health Organization, the number of cancer cases in 2018 increased to 18.1 million new cases and 9.6 million deaths. The leading types of new cancer cases globally are lung, female breast, and colorectal cancers [1]. Therefore, the ability of early cancer detection and treatment is of utmost importance. There are several well-known standard techniques for cancer treatment such as surgery, radiation, and chemotherapy [2,3]. However, these methods cannot efficiently fulfill the need in cancer disease treatment due to the several limitations such as the hard-to-reach tumor position, the close location of other possible tumors, the patient’s opinion, and health conditions. Moreover, cancer tumors can create protection from numerous chemotherapeutic agents, causing additional obstacles for the treatment [4]. This review paper focuses on thermal ablation therapy to outline the recent advances in cancer treatment by use of radiofrequency, microwave, laser ablation, and photothermal ablation sources supplemented by various nanomaterials. Moreover, the advantages and properties of each nanomaterial, namely magnetic and gold nanoparticles, nanocomposites, nanoshells, nanorods, carbon nanotubes, and other types of nanoparticles are discussed. A schematic representation of several types of nanomaterials used is shown in Figure 1. 

**Figure 2:**
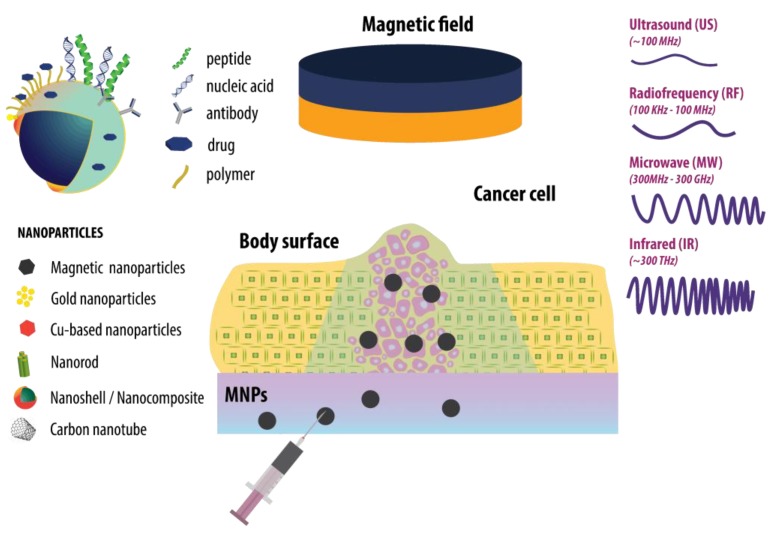
Schematic representation of cancer thermal therapy using the combination of nanomaterials with various surface functionalization possibilities and different external heat sources.

Thermal ablation is a technique used in cancer therapy to eliminate damaged cells or tissue by applying external electromagnetic waves and elevated heat. Thermal ablation techniques utilize radiofrequency, microwave frequency, and cryoablation, and is focused on ultrasound (US) and laser light [5,6]. The advantages of thermal ablation therapy over the conventional methods are the flexibility, low cost, and its minimal invasiveness [7]. However, the choice of a suitable heat delivery route to the tumor is a vital and challenging concern in thermal ablation [8]. Moreover, existing heating methods have difficulties in differentiation between tumors and surrounding healthy tissues, leading to the damage of the neighboring cells [9]. Therefore, the combination of nanotechnology and thermal therapy has attracted a lot of attention as a promising method to overcome the relevant limitations of conventional thermal therapies. A schematic representation of nanomaterials and external heat sources used is shown in Figure 2. 

**Figure 3:**
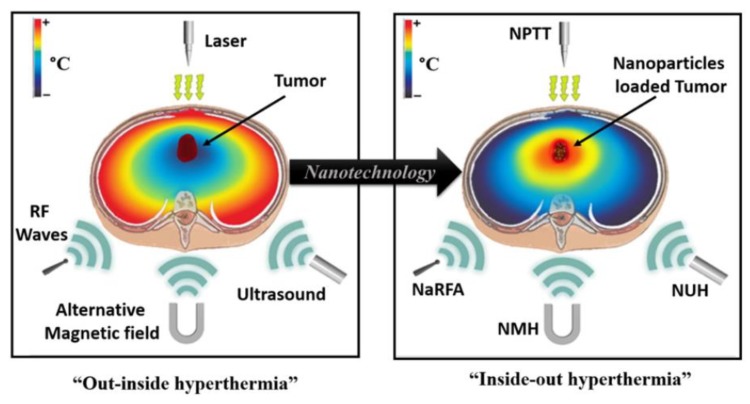
Thermal hypothermia of a tumor using nanoparticles enclosed inside of it absorbing the energy coming from different heat sources. NPTT—Nano-Photothermal Therapy, NMH—Nano-Magnetic Hyperthermia, NaRFA—Nano-Radio-Frequency Ablation, NUH—Nano-Ultrasound Hyperthermia [Reproduced from [8], with permission from Elsevier, Copyright 2016].

Nanotechnology is gaining great attention in the biomedical field due to the possible application in diagnostics and treatment techniques [10,11,12,13]. Nanomaterials, especially nanoparticles-assisted and nanocomposites-assisted thermal therapy, offer many advantages over conventional methods. Due to their magnetic and optical properties, nanomaterials can trigger heat increase in tumor regions by absorbing near-infrared light (NIR), electromagnetic, or radio frequency (RF) waves [14,15]. Moreover, surface-functionalized nanomaterials can specifically bind to the cancer cell and allow selective heat destruction of the tumor and also the multitasking possibility of cell separation and imaging [16,17]. As shown in Figure 3, the nanomaterials allow heat increase at the specific region and prevent the heat generation in the non-targeted region, improving the selectivity of the treatment.

A general procedure for the RF thermal ablation combined with nanomaterials consists of the following stages: the specific and selective attachment of nanoparticles with cancer, validation of binding, and heat administration to the specific area by applying an electromagnetic field [18]. However, in order to have safe tumor destruction, parameters such as the critical heat dose, application of proper magnetic material concentration, and its biodistribution are vital [19,20,21]. In addition, the further removal of the nanomaterial from the targeted area should be considered carefully in order to avoid the undesired heating of non-target tissue [20]. 

**Figure 4:**
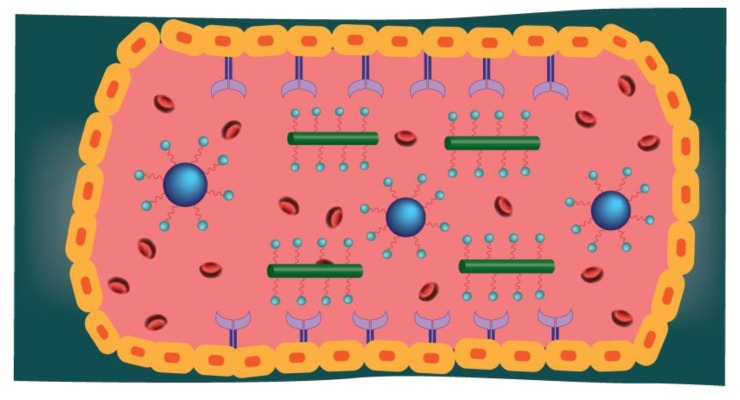
Effects of shape on nanoparticle flow. Spherical nanoparticles move toward the center of the flow, while the rod-shaped nanoparticles tend to attach to wall receptors due to the different forces and torques exerted on rods.

The size and shape of nanoparticles are critical factors that determine their performance: the ability to penetrate the blood vessel, reach the targeted region, affect the rate of macrophage uptake, and finally wash out from the body. For instance, nanoparticles larger than 10 nm will be too large to pull out from the normal capillaries [22,23]. In addition, the transport of smaller nanoparticles exhibits relatively higher diffusion rates, which allows them to move laterally in the blood vessel with greater ease. However, larger particles can penetrate the tumor through the gaps between the endothelial cells in leaky tumor vasculature and remain there for a long time. This phenomenon called the enhanced permeability and retention (EPR) effect. Mainly, the EPR effect is beneficial to deliver the nanoparticles loaded with the drug. The larger nanomaterials can permeate and accumulate at the tumor site for a long time, causing the effective treatment and avoiding the side effects that are due to failure to penetrate the normal tissue. The drawback of the EPR effect is the inhomogeneity of interstitial holes, which affects the uniform penetration and distribution of nanoparticles within the tumor [24,25]. Nanoparticles of different shapes have a different active fractional area, which results in variability in binding avidity, circulation in the blood, and the ability to bind to wall receptors (Figure 4). Thus, the nanoparticle shape affects the rate of tumor deposition and therapeutic efficacy [23].

Moreover, the surface-chemical properties of nanoparticles and their surface coating play a crucial role in further attachment to the cancer cells. Notably, the functional groups on the surface of the nanoparticle are considered as the defining factors of solubility, interaction, and attachment to the cell. Depending on the surface coating, nanoparticles can be defined as positively or negatively charged. Positively charged nanoparticles are the most beneficial in the passage of cell-membrane barriers, and concentrate in the cytosol or nucleus [26]. 

The control of heat delivery and dose to the cancerous cells is significant in order to meet the clinical requirements. The specific absorption rate (SAR) was found as the main factor to estimate the heating of the tissue generated by the magnetic induction. This parameter is proportional to temperature increase, which is defined as the electromagnetic energy absorption rate by a unit mass of biological material, and is defined as follows as in Equation (1), where *λ*—the thermal conductivity of the tissue, *c*—the concentration of nanoparticles, ∆*T*—the required temperature increase, and *R*—the radius of the spherical tumor [27,28].

(1) $$SAR=\;\frac{\Delta \;T\times 3\lambda }{c\times R^{2}}$$

Sapareto and Dewey [29] studied the thermal dosimetry of thermo-therapies, the effect of high temperature, and the duration of exposure in cancer cells. This model is now commonly used in electrical thermo-therapies such as Radiofrequency ablation (RFA) and Microwave ablation (MWA) [30]. Laser ablation studies are often performed on phantoms that mimic the absorption properties of lasers in the infrared [31]. Most phantoms are based on agar jelly with blackened colorants that are designed to match the absorption and anisotropy coefficients of human tissues [32]. Nowadays, colorimetric models are also used in RFA and MWA in order to have phantoms that are able to yield a different color as a function of the peak temperature achieved during rapid ablation phenomena. A recent work has been presented by Mikhail et al. [33], who reported a thermo-chromic phantom for RFA made of polymeric materials.

Beik et al. presented a review of breakthroughs in nanotechnology for hypothermia cancer therapy. The paper presented the results in nano-photothermal therapy, nano-radio-frequency ablation therapy, nano-US hypothermia therapy, and nano-magnetic hyperthermia therapy employing magnetic and gold nanoparticles [8]. The focus of Beik et al. was on radiation sources rather than nanoparticle types. Abadeer et al. [34] conducted a review on the recent progress in cancer thermal therapy using gold nanoparticles. This work included in vitro and in vivo studies and the recent advances of gold nanoparticle photothermal therapy toward clinical cancer treatment. The potential of RF hyperthermia using gold nanoparticles has been investigated and demonstrated as effective under certain conditions, and the treatment may merit further study. Many researchers have also demonstrated effective cancer treatment, at least in the lab, with photothermal therapy [34]. Day et al. reviewed the application of magnetic nanoparticles, nanoshells in near-infrared photothermal therapies, magnetic fluid hyperthermia, and RF ablation [9]. 

The major difference of this review paper compared to others is in the extended investigation of frequently used nanomaterials (magnetic nanoparticles, gold nanoparticles, CuS nanoparticles, nanorods, carbon nanotubes, nanoshells, and nanocomposites) applied in the cancer thermal therapy using radiofrequency (RF), microwave (MW), photothermal and laser therapy.

## 2. Magnetic Nanoparticles (MNP)

**Figure 5:**
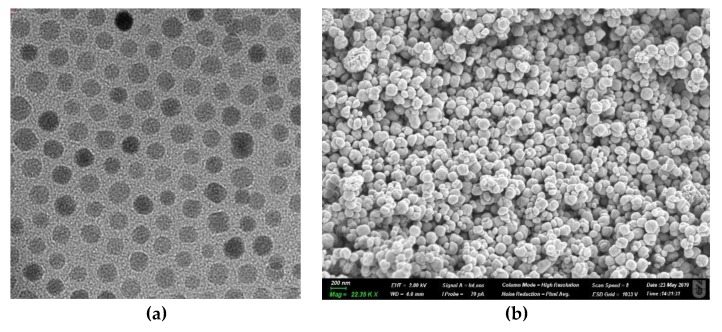
(**a**) The TEM image of iron oxide magnetic nanoparticles (MNPs) synthesized by the thermal decomposition method; (**b**) The SEM image of the iron oxide magnetic nanoparticles synthesized using a solvothermal method.

MNP is a widely used nanomaterial type in thermal therapy with a size range between 1–100 nm. MNPs comprise magnetic elements, such as cobalt, manganese, nickel, iron, chromium, calcium, and gadolinium. Among various MNPs, the iron oxide nanoparticles are widely used in thermal ablation therapy due to the high value of the magnetic moment density, which is 220 emu/g. Moreover, iron oxide MNPs are biocompatible compared to other MNPs, which are known as toxic and susceptible to oxidation [35]. Figure 5 shows that MNPs are mostly spherical in shape when analysed in Transmission Electron Microscopy (TEM) and Scanning Electron Microscopy (SEM).

**Figure 6:**
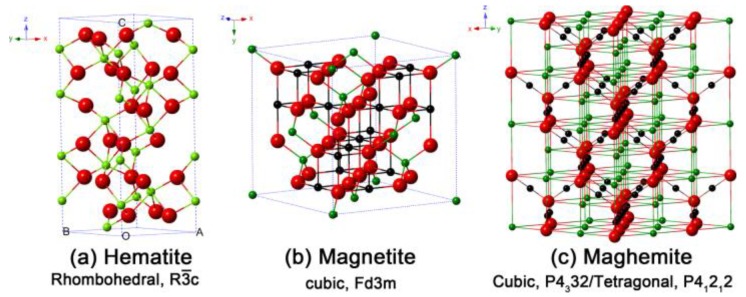
Crystal structure and crystallographic data of the hematite (**a**), magnetite (**b**), and maghemite (**c**) (the black ball is Fe^2+^, the green ball is Fe^3+^, and the red ball is O^2^). [Reproduced from [38], with permission by license CC BY 3.0, Copyright 2015].

Iron oxide nanoparticles according to their structure are classified as hematite (α-Fe_2_O_3_), maghemite (γ-Fe_2_O_3_), and magnetite (Fe_3_O_4_) nanoparticles (Figure 6). Nanoparticles made up of ferromagnetic materials and less than 10–20 nm in size display a unique type of magnetism, which is called superparamagnetic [36]. The phenomenon of superparamagnetism involves magnetization up to saturation magnetization when an external magnetic field is applied, and can return to a nonmagnetic state when the external magnet is removed. This property of nanoparticles occurs in single-domain magnetic particles and depends on the size, which should be as low as 10–30 nm [37].

Among iron oxide nanoparticles, Fe_3_O_4_ and γ-Fe_2_O_3_ exhibit ferrimagnetism at room temperature, leading to the possibility of application in magnetic thermal therapy [38]. The magnetite particle has an inverse spinel configuration with the tetrahedral A-sites and the octahedral B-sites. The A-sites of magnetite structure are occupied by Fe^3+^ ions, and the B-sites are occupied by Fe^2+^ and Fe^3+^ ions. This explains the idealized magnetic moment of Fe_3_O_4_, which is 4 μB per formula unit. The structure of MNPs is one of the key aspects that makes the extensive adaptation of magnetic thermal ablation possible [39].

**Figure 7:**
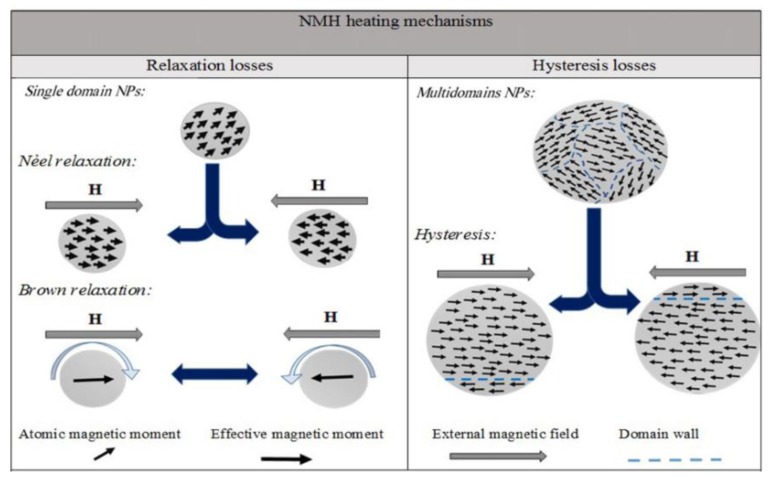
The Neel relaxation and Brownian mechanism representation of heat generation via MNPs under the atomic magnetic moment [Reproduced from [8], with permission from Elsevier, Copyright 2016].

MNPs can produce heat by four distinct mechanisms: eddy currents and hysteresis losses in particles with sizes greater than 1 micron, relaxation losses in superparamagnetic particles, and frictional losses in suspensions that are viscous [40,41]. Relaxation losses can be defined by two mechanisms: the Neel relaxation mechanism and the Brownian motion of the particles. The Neel relaxation mechanism is a rotation of the magnetic moment within a nanoparticle, which remains fixed. The dissipation of energy during the Neel relaxation occurs by rearranging of the atomic dipole moment in crystal. While in Brownian motion, the entire nanoparticle rotates within its surroundings, dissipating the thermal energy to the ambient media through shear stress (Figure 7). The dissipation of magnetic energy of nanoparticle into thermal energy is reasoned by the property of particles to change the orientation of the magnetic moment to align with the field and return to the equilibrium position when an alternating magnetic field (AMF) is applied [42,43,44].

The first groundbreaking research in the utilization of magnetic materials for the enhancement of the thermal therapy was done by Gilchrist et al. in 1957. They experimentally demonstrated on a dog’s lymph nodes that the field strength of 200 to 240 Oersteds with 5 mg of 20–100-nm sized Fe_2_O_3_ nanoparticles per gram of tissue could increase the temperature by 14 °C in three minutes by applying RF heating [45]. Later, in 1965, the same group obtained the elimination of metastasis at 50 °C temperature for 30 min in 15 dogs utilizing the ferrite magnetite nanoparticles that were between 20–100 nm µm [46]. After these studies, many investigations in these field have been done to optimize cancer treatment techniques.

MNPs can be produced by different chemical and physical synthesis techniques, which can influence the final parameters of the nanoparticles [47]. Notably, the method of nanoparticle synthesis affects the final size, shape, and/or surface charge of the final product. The size of MNPs is one of the significant parameters in the thermal therapy. As the size of nanoparticles decreases, the surface-to-volume ratio increases, offering a wide range of optical, electronic, chemical, and magnetic properties [48]. Furthermore, MNPs with small sizes and high surface areas are biocompatible, biodegradable, chemically stable, easy to synthesize, and cost-effective [49,50]. Superparamagnetic iron particles with core diameters less than 6 nm are not able to generate heating properties. Therefore, it is preferred to have particles with a size larger than 10 nm to reach the cytotoxic effects within target cells and the possibility of heat increase [51]. Moreover, Tong et al. in their study defined that MNPs with sizes larger than 15 nm have better heating efficiency. However, the size limits up to 40-nm MNPs, because at this point, the SAR value reaches the theoretical maximum of clinically significant AMF [52]. Many studies employed MNPs that were 10–20 nm to investigate the heat increase at the tumor site [20,51,53,54,55,56,57,58]. In the study of Hilger et al., three MNP samples with 10-nm and 220-nm sizes were employed in the thermal therapy; the maximum temperature of 73 °C was obtained by 10-nm MNPs, while the 220-nm MNPs could increase the heat up to only 50 °C [20].

MNPs found wide application in the treatment of breast, prostate, glioblastoma, kidney, etc. Most researchers concentrated their study on specific cell types. For instance, Hilger et al. focused mainly on the thermal ablation therapy of breast cancer employing magnetic nanoparticles, and demonstrated the possibility of electromagnetic heating of breast cancer tumors with volumes of approximately 300 mm^3^, utilizing 10 female immunodeficient mice and injecting intratumorally 50–100 µL of magnetite fluid at a flowrate of 50 µL/min. For the magnetic thermoablation of tumors, the mice were exposed to an AMF (frequency, 400 kHz; amplitude, 6.5 kA/m) for 242 s. Over the treatment, a mean temperature of 71 ± 8 °C and a maximum temperature of 79 °C were recorded [53]. In another in vivo study by the same group, the thermal ablation procedure of breast cancer using a greater number of mice—exactly 45 mice divided into three iron oxide MNP models by size—was conducted to observe the deposited heat dosage on cell death at different regions of the tumor. The experimental set up demonstrated the higher temperature increment at the center of the tumor (73 °C) compared to the periphery region (12 °C) when applying an alternating current at a frequency of 400 kHz and amplitude of 6.5 kA/m, leading to the conclusion that magnetite nanoparticles mostly concentrated at the tumor center [20]. Another in vitro study of breast cancer treatment using MNP was performed by Kettering et al., concentrating also on BT-474 cells [51]. 

Johannsen et al. conducted a broad investigation of the thermal ablation of prostate cancer tumor, specifically MatLyLu (the R3327 Dunning tumor cell) tumor model using MNPs [56,57,58]. In phase I clinical studies, Johannsen et al. demonstrated the increase of temperature up to 55 °C. However, in some patients, the temperature was recorded as 44 °C at the skin. This research showed the possible clinical challenges of thermal therapy using MNPs. The difficulties of prostate cancer treatment are explained by the high perfusion of this organ, restrained obtainable temperatures, and thermal homogeneity. Adding to these reasons, the prostate is concealed anteriorly by bone and fat and encompassed by hollow organs enclosing fluid and air. The various conductivities of these tissues to RF or US waves utilized for heating objectives can trigger unwanted reflection, scattering, or the absorption of thermal energy outside the target region in critical areas [57]. 

An in vivo study of thermal therapy of glioblastoma multiforme of rat glioma cells (RG-2 cells) using MNPs in 120 rat models was proposed by Jordan et al. Glioblastoma is mainly a brain tumor with high death and low survival rates [55]. The difficulties of brain tumor treatment caused by the several challenges: the location of tumor can be hard to reach, the brain has unique biological features being sensitive to neural mechanisms, and the tumor can be positioned behind the blood–brain barrier [59,60]. Another research group clinically investigated the efficacy of the thermal therapy in combination with radiotherapy in a phase II study of 66 patients with recurrent glioblastoma multiforme using 112 mg/mL of 12-nm amino-silane coated MNPs. The three-month post-treatment monitoring of patients showed an increase in the overall survival rate [61].

Bruners et al. conducted research on the computed tomography (CT)-guided magnetic thermoablation of animals with kidney tumors using a rabbit VX2 carcinoma model in vivo and colloidal superparamagnetic magnetite particles. Exposure to the electromagnetic field was maintained for 15 min with the value of frequency of 56 kHz, inducing an electromagnetic field with a strength of about 0.32 kA/m. The CT-guided injection of nanoparticles demonstrated the inhomogeneous distribution of the injected ferrofluids, and the optimal method of ferrofluid application is still under investigation [62].

Coating the surface of the nanoparticles by functional groups provides a great opportunity for the application of MNPs in biomedicine, namely in drug delivery, cell sorting, separation, tissue repair, and magnetic resonance imaging [51]. Depending on the cancer type, the nanoparticles can be functionalized with antibodies, DNA, polymers, proteins, liposomes, or other ligands to attach specifically and selectively to the tumor region. Moreover, the surface coating prevents the agglomeration of nanoparticles and allows reducing the toxicity of bare nanoparticles [63]. Hilger et al. conducted the research for the in situ and multi-focal treatment of breast cancer tumors to observe the cell death rate during thermal ablation when magnetites and maghemite coated with dextran were used. This research showed that an intratumoral application of MNPs with an iron concentration up to 107 pg/cell could raise the temperatures up to around 71 °C at the center of the tumor in 242 s at a frequency range of 400 kHz and 6.5 kA/m field amplitude. Moreover, the accumulation and attachment of dextran-coated nanoparticles to the breast cancer can be monitored by magnetorelaxometry or—in the case of an in vivo study—by magnetic resonance tomography. This research demonstrated that a multi-focal tumor causes higher complexity during thermal ablation and requires further investigation [54]. Kettering et al. utilized the MNPs with a starch coating to label with the adherent invasive ductal breast carcinoma cell line BT-474 cells at 0.32 mg Fe mL^−1^ concentration. The heat elevation in cells labeled with MNPs was checked by varying the following parameters: a various concentration of cells (0.5, 1, 2.5 and 5 × 10^7^ BT-474 cells mL^−1^), different incubation times (1, 3, 6, 12, and 24 h) and varied concentrations of nanoparticles (0.16, 0.32 and 0.48 mg Fe, respectively, per mL of culture medium for 24 h). The MNP-labeled cells were exposed to an AMF with a frequency range of 400 kHz and an amplitude of 24.6 kA m^−1^. The highest temperature value of 28.2 ± 0.4 °C was observed when cells were labeled with 0.32 mg Fe mL^−1^ culture medium for 24 h and a concentration of 2.5 × 10^7^ BT-474 cells mL^−1^. The research demonstrated the possibility of increasing the concentration of MNPs at the tumor region by labeling with cells through surface functionalization [51]. 

Jordan et al. performed the in vivo thermal therapy of 120 male rats with RG-2 glioma cells at a frequency of 100 kHz and a variable field strength of 0–18 kA/m using MNPs with two types of coatings—carboxydextran-coated and amino-silane coated—in order to observe the relevance of the surface coating on intratumoral temperature homogeneity. The nanoparticles with two types of coatings demonstrated different performances regarding their distribution within the specific region, leading to distinct temperatures for intratumor therapy. Amino-silane coated nanoparticles exhibited homogeneous distribution in the tumor area during heating procedures, and the intratumoral temperatures reached 43 °C and 47 °C by altering the magnetic field strength. The dextran-coated nanoparticles demonstrated an insignificant increase in temperature of only 2 °C [55]. Another study using amino-silane coated MNPs for the thermal therapy of glioblastoma multiforme also demonstrated positive results and a higher survival rate [61].

Johannsen et al. in 2005 proposed an in vivo study of the magnetic fluid thermal ablation of prostate cancer MatLyLu cells using magnetic ferrofluids with an average particle core size of 15 nm that were coated with an amino silane-type shell in water. Compared to previous studies, this research demonstrated an increase of temperature above 70 °C and a significant reduction of local tumor growth in the orthotopic MatLyLu tumor model of prostate cancer [58]. Later in 2007, the same group demonstrated the possibility to achieve thermoablative temperatures with 15-nm core and amino-silane coated MNPs in the prostates utilizing low magnetic field strengths. The nanoparticles with a concentration of 112 mg/m in aqueous liquid were injected transperineally and exposed to the AMF with the frequency range of 100 kHz, and field strength varied from 2.5 to 18.0 kA/m. This research defined an approach for a non-invasive temperature evaluation strategy using distributions of MNPs to predict the distribution of a three-dimensional intraprostatic temperature in each patient [56]. The further preclinical studies proposed the contactless, selective heating of superficial tumors on 10 patients with biopsy-proven, locally recurrent prostate cancer. The amino-silane-type shell-coated nanoparticles with an average core size of 15 nm were injected transperineally into the prostates. Six thermotherapy sessions were delivered at weekly intervals using an AMF with a frequency of 100 kHz and variable field strength (2.5–18 kA/m). The maximum temperatures reached 55 °C at 25–30% of the magnetic field strength available [64]. Balivada et al. demonstrated the antitumor potential of the modified bimagnetic core-shell of stealth-coated dopamine-labeled Fe/Fe_3_O_4_ nanoparticles with the size of 7.2 ± 2.8 nm intratumorally injected on murine B16-F10 melanoma with three short 10-minute AMF exposures in mice over 14 days. This study revealed a significant decrease in tumor weight using the Fe/Fe_3_O_4_ nanoparticles [65].

The distribution of nanoparticles within the tumor area is a crucial point in thermal therapy in order to obtain homogeneous heating. Richter et al. proposed the use of magnetorelaxometry (MRX) as an appropriate instrument to monitor the MNP distribution quantitatively with less contact. In another study, 200 µL of a 200-nm MNP suspension was injected into the tumor of female Severe Combined Immune-Deficient (SCID) mice. The quantification results by multichannel MRX show that 24 h after intratumoral MNP injection, all the administered MNPs were found in the tumor site. This research demonstrated the possibility of quantitative controlling the distribution of distribution within the tumor area during the magnetic ablation [66]. Finally, Andra et al. performed spatial temperature distribution by means of a mathematical simulation. This research demonstrated an experimental study satisfying the numerically simulated studies at 6.5 kA/m amplitude and 400-kHz frequency of the applied field. However, the results were restricted to small tumors [67].

Table 1 presents a summary of MNPs application in thermal ablation therapy, demonstrating the general ablation conditions, type of cancerous cells and the increase of temperature.

## 3. Gold Nanoparticles (AuNP)

**Figure 8:**
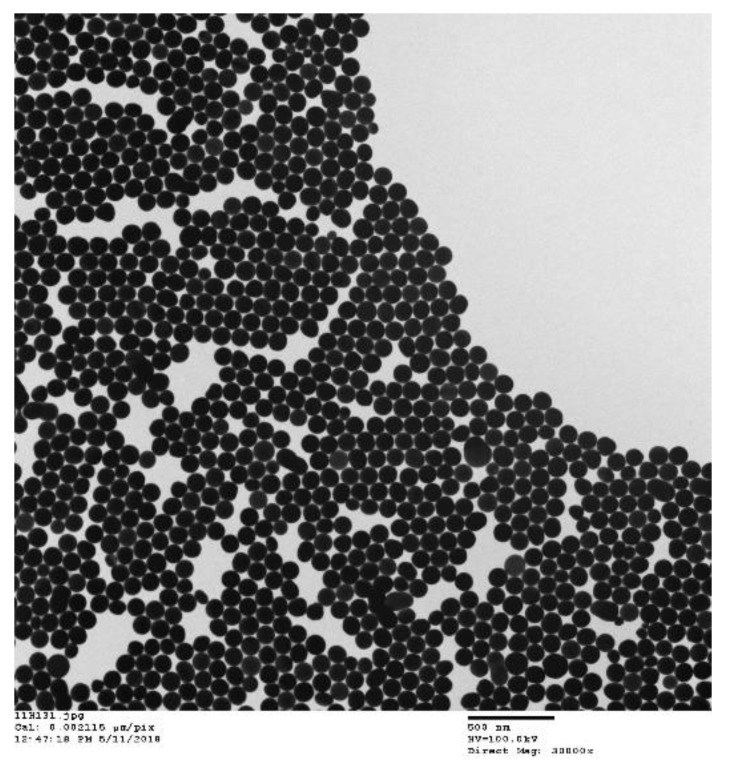
A TEM image of spherical gold nanoparticles (AuNPs), provided by Nanopartz Inc company.

In recent years, AuNPs are actively employed in biomedical applications. AuNPs are known as the most stable metal nanoparticles and can be easily synthesized in different shapes [68]. In addition, AuNPs have tunable optical properties, flexible surface chemistry, and a broad range of functionalization possibilities by antibodies, peptides, and polymers for application in various fields. Moreover, one of the main advantages of AuNPs is the ability to convert the light or RFs into heat, owing to the surface plasmon resonance phenomenon of gold, which enables their use in thermal ablation therapy [69,70,71]. A TEM image of spherical AuNP is presented in Figure 8. 

AuNPs are mainly used in the thermal treatment of cancer, employing RF ablation and laser photothermal ablation. The distinction between conventional hyperthermia and photothermal therapy is that during photothermal heating, the increase of heat occurs around the AuNPs, leading to the rise of local temperatures to tens or hundreds of degrees above physiological temperature [9]. 

The size of AuNPs used for thermal ablation varied between 5–40 nm, depending on the type of ablation and cell line. Smaller AuNPs are preferred in cancer thermal therapy because light is absorbed by nanoparticles, which affects the valuable cell destruction when converted to heat [69]. Moreover, smaller particles have higher surface area-to-volume ratios, providing more opportunities for biological elements or proteins to attach to the particles’ surfaces. Finally, the smaller particles can be easily removed from the human vasculature. The shape, cell type, and surface coating along with the size are vital parameters for the cancer cells’ destruction using AuNPs. This review presents the use of AuNPs in the treatment of epithelial carcinoma, liver cancer with hepatocellular (HepG2 and Hep3B) cancer cell lines, and pancreatic cancer with a human pancreatic cancerous cells (Panc-1) and human pancreatic ductal adenocarcinoma cell line (Capan-1) [69,72,73,74,75,76].

Notably, the possibility of gold surface functionalization is beneficial to differentiate between cancerous and healthy cells to specifically attach the AuNPs to the desired tumor region. Furthermore, the coating of the AuNPs’ surface is important in order to make the nanopartciles selective to the specific cancer and effective in thermal treatment, and also to limit their toxicity level. One of the frequently used ligands to conjugate with AuNPs and employ further in thermal therapy is an anti-EGFR antibody (epidermal growth factor receptor), which is responsible for the cell transduction mechanisms in ordinary and cancer cells. The choice of anti-EGFR antibody conjugation for the labeling of cells with AuNPs is reasoned by EGFR overexpressing in most tumor cells [77]. In the study performed by El-Sayed et al., the laser photothermal therapy of the epithelial carcinoma HaCaT cells (human keratinocyte cell line) was carried out using an anti-EGFR antibody conjugated to AuNPs with the average particle size of 40 nm at 530-nm absorption maximum. The photothermal stability of AuNPs was investigated by exposing the cells to the different densities of the laser power such as 64, 57, 51, 45, 38, 32, 25, 19 and 13 W/cm^2^ at various points for 4 min. This research showed that the HaCaT cells after incubation with the anti-EGFR antibody-conjugated AuNPs can cause the complete destruction of the tumor at a laser density higher than 57 W/cm^2^ compared to control groups of two oral squamous carcinoma cell lines (HSC 313 (Hematopoietic stem cell) and HOC 3 (Cellosaurus cell line) ). The study demonstrated not only the importance of the nanomaterial surface functional groups, but also the value of the laser power density [72].

The research conducted by Huang et al. also demonstrated the beneficial selective kill of cancerous cells using AuNPs coated with anti-EGFR. The laser photothermal therapy of cancerous cells showed the death of cancerous cells at much lower laser powers than those needed for healthy cells—200 nW compared to 750 mW, respectively—and less dosage requirement. The study showed that a threshold temperature in the range of 70 to 80 °C is required to destroy cancerous as well as noncancerous cells, which complies with the data of previously conducted research studies [78]. Another study with 20-nm sized anti-EGFR functionalized AuNPs was performed by the group of Glazer et al. for the RF heating (13.56 MHz, 200 S, 10−15 kV/m, 5 min) of pancreatic cancer. The research investigated the importance of surface chemistry by comparing the cell lines overexpressing and not expressing the EGFR, which are Panc-1 and Cama-1, respectively. Finally, a 61% decrease in viability was observed for Panc-1 compared to the control Cama-1 cell line, which reached only 6.3% decrease [76]. 

A further clinical in vivo investigation of the thermal therapy of pancreatic cancer was done by the same group,where two human pancreatic carcinoma cell lines were exposed to the RF field, Panc-1 and Capan-1, utilizing PAM4 hemi-antibody conjugated and C225 antibody conjugated with 20-nm sized AuNPs. The hydrodynamic diameter of the C225-AuNP is 32.6 ± 0.7 nm, while it is 36.9 ± 1.5 nm for PAM4-AuNP. C225-conjugated AuNPs have been more efficient in destroying targeted tumors than PAM4-conjugated AuNPs. However, C225 as a targeting agent can have more negative clinical occurrences due to the increased absorption of AuNP in non-malignant tissues. PAM4 appears to be much more specific to pancreatic cancer, and only works as a pancreatic adenocarcinoma that targets an antibody without an intrinsic cytotoxicity or growth inhibition characteristics [75].

The cytotoxicity effects of AuNPs was also investigated by other research group on non-invasive RF ablation hepatocellular (Hep3B) and pancreatic cancerous cells (Panc-1) at 13.56 MHz employing 5-nm sized AuNPs with 67 µM/L concentration. The cytotoxicity parameter is vital for the further use of nanoparticles in clinical purposes. The authors reported the absence of the intrinsic cytotoxicity of AuNP, and the range of malignant cell destruction was 99.8 ± 3.1% for Hep3B cell death and 96.5 ± 8.4% for Panc-I cell death in 5-min exposure [74]. 

The significance of RF field power on thermal therapy was explored by Cardinal et al. This group studied the non-invasive RF ablation of liver cancer with HepG2 cancerous cells employing 13-nm citrate coated AuNPs. To determine the effect of RF field power on heating, solutions were exposed to the RF field at variable powers from 10 W to 100 W for 3 min. The obtained data demonstrated the ability of heat elevation for more than 50 °C by AuNP-containing solutions. Moreover, after 7 min of heat exposure, 80% of cell death occurred [73].

An overview of AuNP application and conditions in thermal therapy of cancer is presented in Table 2. 

## 4. CuS Nanoparticles

Semiconductor copper sulfide (CuS) nanomaterials display good optical, electrical, and catalytic properties by absorbing the infrared light from energy band–band transitions. CuS nanoparticles found wide application in biological labeling, the detection of DNA, eye protection, monitoring the laser light, and the photodegradation of pollutants. CuS nanoparticles are promising due to their minimal cytoxicity effects, which is similar to the gold nanoparticles, and low cost [79]. The minimal cytotoxicity of CuS nanoparticles is explained by the slow rate of dissociation rate of Cu ions [80].

The CuS nanoparticles are mainly used in the photothermal therapy of cancer with the size range as low as 3 nm. Li et al. were the first to study the photothermal ablation of cancerous cells utilizing the heat generated by the interaction of CuS nanoparticles when NIR light was applied in 2010. The research was performed using 3-nm uniformly distributed thioglycolic acid-stabilized CuS nanoparticles with a density of 4.6 g/cm^3^ for the photothermal ablation (PTA) of Human cervix adenocarcinoma (HeLa) cells. The intensive absorption of the NIR by CuS nanoparticles enables their use in PTA therapy. When CuS particles with the concentration of 770 µM (approximately 1.42 × 1014 particles/mL) labeled cell lines were exposed to a laser beam at 808 nm with the output power of 24 W/cm^2^, the temperature increased by 12.7 °C and reached 37 °C in 5 min. No changes were observed when water was used at the same conditions. This research demonstrated the dependence of cell death on the nanoparticles concentration and the laser’s output power. Moreover, in comparison with nanostructures, CuS nanomaterials offer several advantages. First, CuS is cheaper than gold. Second, the absorption wavelength of CuS nanomaterials do not depend on the particle size, shape, or solvent due to the NIR absorption in CuS arising from the d–d transition of Cu^2+^ ions, while in AuNPs, the NIR absorption occurs from the surface plasmon resonance. Finally, the 3-nm CuS nanoparticles can offer the desired pharmacokinetic properties for targeted delivery. However, the properties of CuS nanoparticles are limited by the low efficacy of the photothermal conversion [79].

Near-infrared light-responsive inorganic nanoparticles can increase the efficiency of cancer photothermal ablation therapy. Moreover, CuS nanoparticles can be used in the combination of photothermal therapy with immunotherapy, as it was proposed in the study of Guo et al. [81]. The advantage of current research consists of the ability to utilize the chitosan-coated hollow copper sulfide nanoparticles (HCuSNPs) with a porous shell structure for the treatment of metastatic cancer. These nanoparticles were composed of small crystals with a diameter of 10 × 2 nm. This research demonstrated that the combination of photothermal therapy and immunotherapy was more efficient for the tumor treatment than when they were used separately. The results of this study were compared to previously published data, where regularly injected pegylated HCuSNPs were eradicated through both renal and hepatobiliary excretion. This study demonstrated a comparatively higher amount of Cu eradication, which is 47% from tumors. This was associated to photothermally activated disintegration of the original ∼85-nm HCuSNPs into ∼10-nm small CuS nanoparticles, providing faster clearance from the tumor [81].

Huang et al. in 2014 investigated the application of 3.8-nm sized hydrophobic copper sulfide nanoparticles with a phospholipid–PEG coating (CuS@DSPE-PEG NPs) for the treatment of cervical cancer with HeLa cells in mice, applying the photothermal ablation. The team incubated nanoparticles with several concentrations to damaged cells and exposed them to an 808-nm laser with a power density of 1.0 W cm^2^ for 8 min in order to investigate their influence on the death rate of cells. A total of 80% cell death appeared at a 400 mg/mL concentration of CuS@DSPE-PEG NPs. Further research was conducted to examine the in vivo photothermal effect of CuS@DSPE-PEG NPs employing the S180 tumor-bearing Kuming mice. A tumor surface temperature above 59.2 °C was achieved promptly within 5 min, while in the control group not containing nanoparticles, the temperature reached about 48 °C in mice [82]. Another study employing PEGylated CuS nanoparticles was conducted by Zhou et al. The research utilized the developed PEG-coated single radioactive copper sulfide (CuS) nanoparticle platform to enhance the efficacy of photothermal therapy (PTT) in a murine orthotopic model of anaplastic thyroid carcinoma (ATC). The investigation of temperature elevation induced by NIR light irradiation was carried out in the presence of PEG-CuS NPs using a continuous-wave fiber-coupled diode laser centered at 980 nm. Exposure to the NIR laser light at 2.5 W/cm^2^ elevated the temperature of the CuS NP solution with 400 mg/mL of 0.1-mm CuS NPs from 23 °C to 98 °C in 10 min. The temperature of pure water was elevated only from 23 °C to 32 °C during the same period. In addition, tissue distribution of polyethylene coated (PEG-[^64^Cu]) CuS NPs could be imaged and quantified by Positron Emission Tomography allowing dosimetry calculation and potential for the prediction of thermal dose [83].

Table 3 provides a summary of CuS NPs size, exposure conditions and temperature increase in the thermal therapy of cancer.

## 5. Nanorods

**Figure 9:**
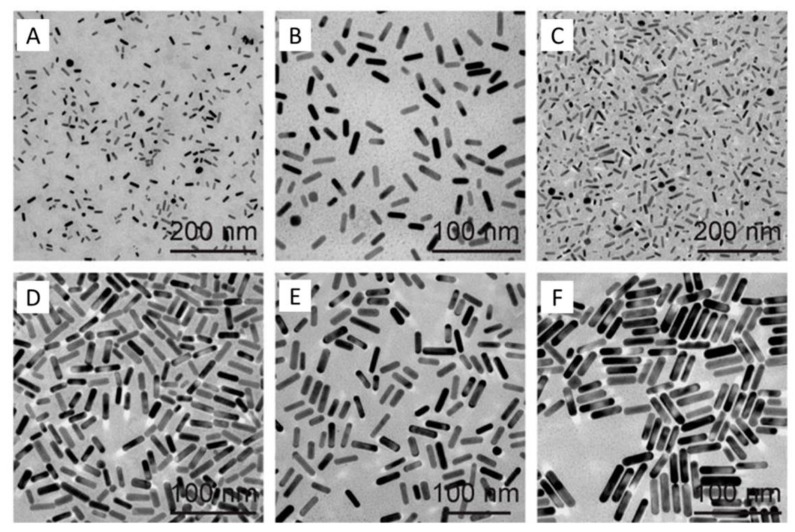
TEM images of gold nanorods samples (GmSn) differentiated by the molar ratio of seed-to-Au(III) in growth media. (**A**–**C**) Gold nanorods were grown with cetyltripropylammonium bromide (CTPAB), and (**D**–**F**) gold nanorods grown with CTAB [Reproduced from [88,89], with permission from Open Access Journal, under the license CC BY 4.0, Copyright 2017].

Gold nanorods are the unique class of metal nanostructures with two surface plasmon absorption long and short wavelength bands. The strong long wavelength band lies in the NIR region due to the longitudinal oscillation of the conduction band electrons, and the weak short wavelength band is around 520 nm because of the transverse electronic oscillation. The surface electric field enlarges because of the surface plasmon excitation, which facilitates strong absorption and the scattering of electromagnetic radiation by gold nanorods. Gold nanorods can be used in biomedicine due to their tunable optical absorption and scattering properties [84,85]. Gold nanorods gained attraction in photothermal therapy due to the possibility of their synthesis with various aspect ratios, which enable selective absorption in the NIR region [86,87]. A TEM image of gold nanorods is shown in Figure 9.

The most commonly used surface coating for nanorods is polyethylene glycol (PEG). The advantages of using PEG in its affinity to cell membranes, leading to a high tumor uptake rate [90]. In addition, PEG is available in different polymer lengths. The nanoparticles coated with PEG are stable, biocompatible, and nontoxic for in vitro application [24]. Von Maltzahn et al. developed long circulating PEG-coated gold nanorods and compared their effects with the efficacy of nanoshells. The authors simulated a therapeutic irradiation regimen based on heat transfer simulations, which are able to successfully plan the therapy to destroy tumors on mice injected with PEG-NRs using half the light intensity of previous nanoshell therapies [91]. 

Huang et al. investigated the effects of gold nanorods in prostate cancerous cells using a CW (continuous wave) laser emitting at 800 nm, 20 W/cm^2^, using different exposure times ranging from 4 min to 20 min. They found an acceptable agreement between the theoretically predicted results and experimental ones [92]. 

The theoretical investigation of the effect of the distribution of nanorods delivered to a skin tumor for the thermal ablation procedure was reported by Soni et al. Three distribution arrangements of gold nanorods with 5-nm diameters and 2.5-mm depth were considered: uniformly distributed within the tumor region, deposited in the tumor core, and accumulated at the tumor periphery area through intravenous injection. In the first case, the temperature increased up to 75 °C at the surface of a tumor. However, the lower temperature change was achieved at the tumor depth, comparatively, at 2.5 mm and 5 mm, for which the temperature was about 48–58 °C and 43–48 °C, respectively. For the second case, the top half-central region of the tumor reached a temperature of 50–65 °C, and the deeper tumor location remained at 37 °C. Finally, the concentration of gold nanorods at the periphery of the tumor area led to a uniform temperature distribution and demonstrated better heat release to the deeper tumor region, reaching the temperature of 48–52 °C. The photothermal irradiation intensity of 1.25 W/cm^2^ for 300 s at a volume fraction of 0.001% were determined as an optimal parameter to obtain the temperature value higher than 53 °C over the whole tumor region [93]. 

Cho et al. proposed the study of photothermal laser ablation using PEGylated gold nanorods conjugated with C-225, which is a recombinant, human/mouse chimeric monoclonal antibody to epidermal growth factor receptor (αEGFR). A fiber-coupled diode laser (DILAS) emitting at 808 nm was used in thermal ablation experiments. The spot size was approximately 5 mm^2^ and the cells were exposed at an irradiation time of 1 minute with varying laser power densities from 20 W/cm^2^ to 90 W/cm^2^ or at 20 W/cm^2^ with varying irradiation times from 30 s to 3 min. Upon reviewing the binding assay results for various Monomethoxy PEG thiol (mPEG-SH) concentrations, 4 mg/mL showed the most uniform fluorescence with the least agglomeration, and was used for the in vitro thermal ablation experiments. Moreover, in the control samples treated with C-225 only, no cell death was observed until the power density was increased to 90 W/cm^2^, which was the highest level used in these experiments. In contrast, samples treated with αEGFRAuNR showed clear trypan blue staining, indicating cell death, at a laser power level as low as 20 W/cm^2^ [94].

**Figure 10:**
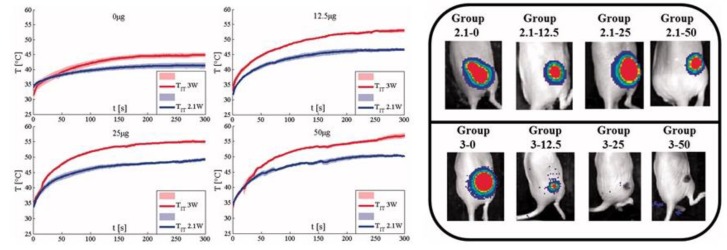
AuNRs-mediated LA in mice bearing MDA-MB-231 human breast cancer. Temperature evolutions measured inside the tumor during ablation performed at 2.1 W and 3 W. Xenogen images acquired two days after ablation [Reproduced from [96], with permission from Taylor & Francis, Copyright 2019].

Mooney et al. in 2015 and 2017 investigated the effects of laser ablation enhanced by gold nanorods. Experiments were performed on mice. Firefly luciferase expressing MDA-MB-231 human breast cancerous cells were injected into the flank of the animals, and the ablation was performed on subcutaneous tumors (growth on the flank of each mouse). The tests were performed at different nanorod masses (0, 12.5, 25, and 50 μg per tumor), laser powers (1.4 and 2 W/cm^2^), and treatment times (0.5, 1, 2, and 5 min). The results showed that the higher the concentration, the higher the temperature increase during the laser ablation. For instance, at 2 W/cm^2^, the control group (without nanorods) showed a temperature increment of approximately 15 °C, while the group treated with 2 W/cm^2^ and 50 μg of nanorods experienced a temperature increment of approximately 23 °C. The temperature increment also influenced the effects of the laser ablation: in the control group, the laser ablation was not successful, while the group with 50 μg of nanorods experienced a full resorption of the tumor (Figure 10) [95,96]. 

Dickerson et al. proposed the study of photothermal therapy of HSC-3 human squamous carcinoma cells in mice by the intravenous injection of PEGylated gold nanorods with dimensions 12 nm in width and 50 nm in length (4.0 aspect ratio), with a longitudinal plasmon absorption maximum at 800 nm. Quick heating occurred upon exposure, followed by steady-state equilibrium, and more than 90% of the observed temperature rise happened within the first 3 min [97]. Jang et al. investigated photothermal ablation using different concentrations of PEG-coated gold nanorods. In each experimental set, the final particle concentration of I gold nanorods n the polyacrylamide phantom was 0.0, 0.1, 0.25, 0.5, 1.0, 2.0, or 5.0 nm. Each gold nanorod-suspended phantom was illuminated with light from an 810-nm diode laser. A relatively uniform distribution of temperature was observed in phantoms with a 5-nm concentration of gold nanorods. Non-uniform temperature distribution along the depth of a phantom was observed in phantoms comprising a higher concentration of GNRs. For instance, the temperature at the top part of the phantoms reached more than 60 °C, while 35 °C was recorded at a distance 6 mm from the surface of a phantom. The difference in temperature at 2-mm and 10-mm distances of illumination was comparatively small at low concentrations of gold nanorods (0.1, 0.25, and 0.5 nm), compared to high concentrations (1.0, 2.0, and 5.0 nm). The study concluded that time and power laser irradiation help to control the temperature in the deep region of a phantom [98]. 

Huff et al. investigated the effect of gold nanorods on adherent human KB cells (the cell line extracted from oral epithelium) thermal therapy by changing the titanium to a sapphire laser. KB cells treated with CTAB-coated nanorods were exposed to continuous wave irradiation for 30 s, at fluences ranging from 15 to 120 J/cm^2^. These cells were destroyed at 30 J/cm^2^ with the harsh bubbling of their membranes after 30 s of irradiation. This study demonstrated that nanorods can be induced at much lower power densities, and cells are not required to be internalized fully to provide photothermal damage [86].

The parameters and conditions of cancer thermal ablation using gold nanorods are summarized in Table 4.

## 6. Carbon Nanotubes (CNTs)

**Figure 11:**
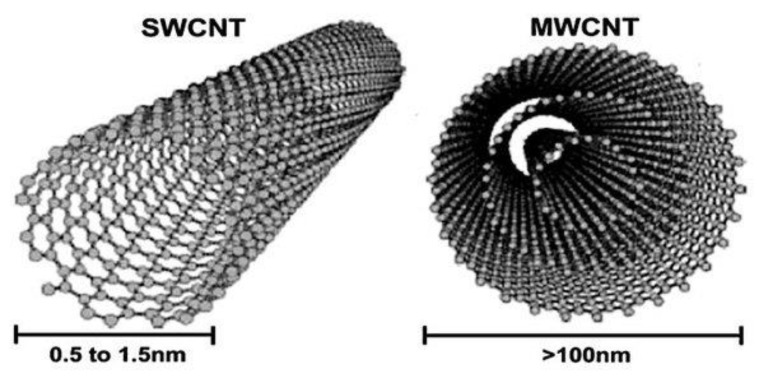
Schematic overview of single-walled nanotubes (SWNTs) and multi-walled nanotubes (MWNTs) [Reproduced from [109] with license CC BY 4.0, Copyright 2017].

Single-walled CNTs are cylinders of nanometer size consisting of a single graphene sheet wrapped up to form a tube (Figure 10) [99,100]. CNTs can be differentaiated as single-walled nanotubes (SWNTs) and multi-walled nanotubes (MWNTs) (Figure 11), depending on the number of graphene layers from which the nanotubes are composed [101]. There are several known CNT synthesis techniques: carbon arc discharge, laser ablation of carbon, and chemical vapor deposition. Nanotube diameters range from 0.4 to 3 nm for SWNTs and from 1.4 to at least 100 nm for MWNTs [102]. CNTs are distinguished by their physical and chemical properties, and have been intensively explored for biological and biomedical applications in the past few years [103]. SWNTs have strong optical absorption properties in the near-infrared (NIR) range [101,104,105]. Single-walled carbon nanotubes generate significant amounts of heat upon excitation with near-infrared light at 700 to 1100-nm wavelengths, which is transparent to biological systems, including skins, and hardly absorbed by normal tissue [106]. CNTs have the opportunity to be employed not only in imaging but also for drug delivery and thermal ablation. This is mainly because of the characteristics of these materials, including their unique chemical, physical, and biological properties, nanoneedle shape, hollow monolithic structure, and their ability to obtain the desired functional groups on their outer layer [107,108].

CNTs can convert NIR laser radiation into heat that is reasoned by the photon–phonon and electron interactions [110]. This property of carbon nanotubes leads to the wide application of them in biomedical application, especially in thermal therapy for cancer. In order to achieve the specific tumor cell targeting, as well as improve the biocompatibility and cell-penetrating capability, the surface of carbon nanotubes need to be functionalized [111,112,113]. 

Gannon et al. discovered the ability of SWNTs to release heat in an RF field, which can be employed to yield the thermal cytotoxicity in tumors. The research was conducted to investigate the treatment of three cell lines such as HepG2 and Hep3B hepatocellular cancer cells and Panc-1 pancreatic adenocarcinoma cells. The cells were incubated at several concentrations (5 mg/L, 50 mg/L, 125 mg/L, 250 mg/L, and 500 mg/L) of biocompatible polymer functionalized SWNTs and exposed to RF ablation at a 13.56-MHz frequency and power value of 800 W for 1 or 2 min. Total (100%) cytotoxicity in all three cell lines was determined at high concentrations of SWNTs (500 mg/L) after 2 min of RF exposure, demonstrating the relevance of concentration [114]. 

The study on breast cancer treatment using carbon nanotubes coated with anti-Her2^+^ antibody was conducted by Marches et al. The research set out to determine the efficiency and selectivity of the NIR-mediated thermal ablation of Her2^+^ human breast carcinoma cancer cells. Cells were irradiated with 808-nm light at 9.5 W cm^−2^ for 4 min. The efficiency of cell killing increased, and was greater than 90% by 24 h. This study demonstrated that Her2+ cells internalized with CNTs were more sensitive to NIR-mediated photothermal destruction compared to the cells with CNTs on their surface [115]. 

The photothermal treatment of colon cancer employing the SWNTs was investigated by Hashida et al. For that purpose, SWNTs were combined with a (KFKA)_7_–peptide composite. The surface-functionalized samples at concentrations of 0.75 μg/mL for colon 26 and 2.5 μg/mL for HepG2 cells, respectively, underwent the NIR laser irradiation. The research demonstrated a high rate of cell death rate and temperature rise up to 43 °C in 30 s for both cell lines [116]. 

Fisher et al. investigated the capability of MWNTs coupled with laser irradiation to increase the tumor treatment temperature and destruction of cells. A human androgen-independent prostate cancer cell line (PC3) and murine renal cancer cell line (RENCA) were laser irritated at 15.3 W/cm^2^ and a 5-mm beam diameter for 1.5 and 5 min using 900-nm MWNTs. Larger temperature elevations were observed for both samples at 5 min of ablation, and reached 43 °C compared to the control sample without MWNTs, where the temperature increase was observed at 7 °C. However, the use of MWNTs caused differences in cell viability [117]. The study by Mocan et al. demonstrated the ex vivo laser-mediated ablation of selectively targeted pancreatic cancer Panc-1 cells using human albumin bound to MWCNTs. The ablation of the tumor was done for 30 min using a 5 W/cm^2^ 808-nm continuous laser generator. The temperature changes in 20 min were recorded as 4.2 °C in the surrounding healthy tissue, 25.6 °C at the periphery of the tumor, and 29.3 °C in the central region of the tumor, showing that the temperature is higher in the center. The high value of temperature that was able to produce extensive tumor necrosis was achieved in 20 min [118]. Table 5 summarizes thermal ablation of cancer using carbon nanotubes.

## 7. Nanoshells/Nanocomposites

### 7.1. Nanoshells

**Figure 12:**
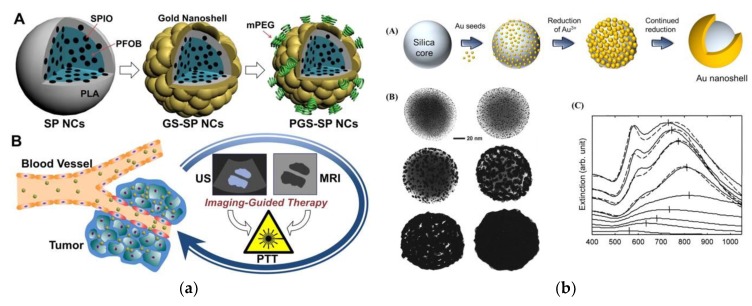
(**a**) Silica core–gold shell nanoshells [Reproduced from [122], with permission from Elsevier, Copyright 2017 ]. (**b**) Silica–gold nanoshells, silica–polymer nanoshells [Reproduced from [123], with permission from the open access journal under the terms of the Creative Commons License, Copyright 2014].

Nanoshells are a class of nanoparticles with 1–20 nm thin coating over the core that can be fabricated of a different material. The properties of nanoshells are possible to adjust by varying the core-to-shell ratio [119,120], as in Figure 12a,b. By varying the thickness of the core and shell, nanoshells can be produced to have light absorbing or scattering properties at the desired wavelength across visible and NIR wavelengths. This explains their optical tunability and makes it possible to fabricate the nanoshells with a peak optical absorption in the NIR [121]. As a NIR absorber, nanoshells are optically very robust; their rigid structure and noble metal surface allow to have the stability to chemical and thermal denaturation and photobleaching effects than conventional NIR dyes [119]. Nanoshells can be accumulated at the tumor area, absorb the NIR light, and produce heat locally to destroy the cancer cells [9].

The most common type of nanoshell composition is silica–gold. The frequently used size of nanoshells are 100–150 nm for the shell and 8–10 nm for the core. The size of nanoshells is explained by the ability to absorb the light in the first and second NIR window. The first NIR window corresponds to 650–850 nm, while the second NIR window corresponds to 950–1350 nm. At these wavelength ranges, light can penetrate the normal cells and reach the nanoshells bound to the tumor [124]. The studies demonstrated that the mainly used wavelength range is 800 nm, because at this NIR window, the light can permeate the tissue securily [119,121,124,125]. 

Hirsh et al. have first proposed the study on the use of silica–gold nanoshells where the core diameter is 110 nm and the gold shell thickness is 10 nm. These nanoshells’ peak absorbance at 820 nm employed for an in vivo study in mice of photothermal carcinoma with breast cancer SK-BR-3 cells treatment under the 35 W/cm^2^ for 7-min laser exposure conditions. The research demonstrated an average temperature increase of 37.4 °C in 4–6 min at the laser dosages that were 10-fold to 25-fold less compared to previous studies [119]. O’Neal et al. proposed the research for the laser treatment of CT26.WT murine colon carcinoma tumor cells in mice using the PEGylated nanoshells with an 8–10-nm thick gold shell over the 110-nm silica core. Nanoshells with 805–810-nm peak optical absorption could increase the treatment temperature up to 50 °C in 30 s, administering 2.4 × 10^11^ nanoshells/mL solution in mice. Within 10 days, the complete resorption of the tumor was observed, while at 90 days post-treatment, all the mice remained healthy [121]. 

Another study on the thermal therapy of cancer cells using the silica–gold nanoshells was carried out by Stern et al. The experiments investigated the treatment of prostate tumors in mice using a 110-nm silica core and 10-nm gold-coated nanoshells by intravenous injection and the application of a NIR laser at 808 nm. Tumors were observed over 21 days and demonstrated 93% tumor necrosis and regression in a high-dose treated group. The temperature was increased up to 65.4 °C. However, limitations exist in quantifying the delivery of nanoshells [126]. 

Bernardi et al. proposed a method for the in vitro photothermal ablation of medulloblastoma and glioma cell lines using anti-HER2 and anti-IL13Ra2 antibody labeled nanoshells (core: 100-nm silica, shell: 10-nm gold) with peak light absorption at 800 nm in the NIR region. The results demonstrated that the implemented approach can selectively kill medulloblastoma cells that express HER2 without killing cells that do not express HER2. In addition, the research showed that the dual requirement for the presence of both nanoshells and laser light to induce cellular death is particularly promising for application in brain tumors [125] 

The application of nanoshells in both imaging and therapy approach has shown promising results for specific cancerous cells. In their in vitro study, Loo et al. demonstrated first detecting by imaging and then thermally ablating human breast cancer cells that overexpress HER2 using immunotargeted nanoshells that have been designed to both scatter and absorb light within the NIR [127]. Schwartz et al. proposed an in vivo study of the minimally invasive treatment of canine prostate cancer in male dogs using 150 nm in diameter spherical gold-over-silica nanoshells with an absorption region that peaks near 800 nm. NIR radiation was provided by a 15-W gallium arsenide diode laser. The results demonstrated the possibility of ablative thermal lesions. Moreover, the study suggested that an optical fiber applicator can have precise tumor ablation [128]. Finally, Xu et al. conducted research on the application of graphitic carbon-coated ferromagnetic cobalt nanoparticles (C–Co-NPs) with diameters of around 7 nm with a 2.5 μg mL^−1^ concentration and cubic crystalline structures for the RF ablation of mammalian cervical cancer cells (HeLa cells). The study demonstrated the destruction of 63% of the cells within 10 min of RF ablation under the 350-kHz and 5-kW parameters [129].

### 7.2. Nanocomposites

**Figure 13:**
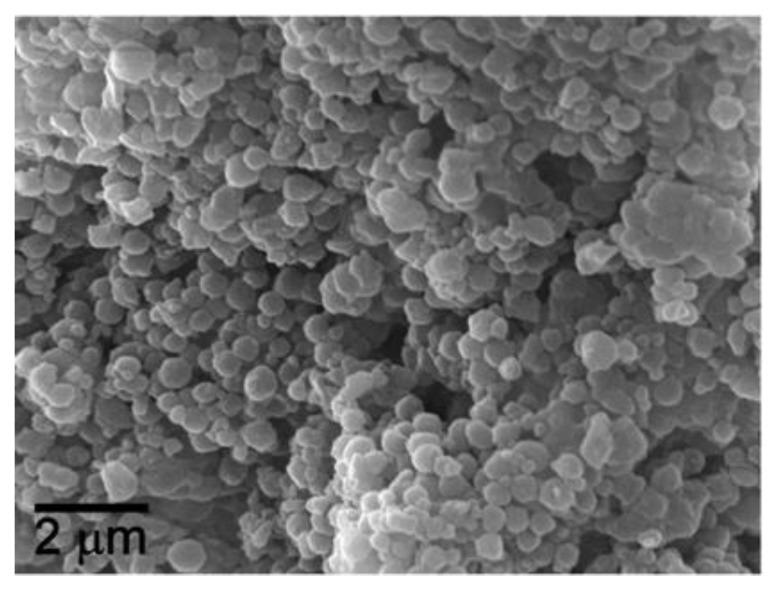
A SEM image morphology of polymer-coated iron oxide superparamagnetic nanocomposite [Reproduced from [130], with permission from Elsevier, Copyright 2008].

The term nanocomposite describes a class of composites consisting of two or more materials, where one of the phases has at least a dimension less than 100 nanometers. Compared to nanoshells, nanocomposites can have micron-sized components (Figure 13).

Xu et al. conducted research on RF treatment of human cancer cell line Panc-1 using highly biocompatible luminescent superparamagnetic nanocomposites Fe_3_O_4_ nanoparticles coated with a silica shell, Fe_3_O_4_/SiO_2_ (IOS), and water-soluble CdSe-ZnS quantum dots (QDs). Panc-1 cells were cultured with various concentrations of nanocomposites and exposed to a frequency of 350 kHz. This study demonstrated the influence of nanocomposite concentration on the rate of cell death. Thus, 0.83 µg/mL nanocomposite could achieve 82.2–83.8% of total cells death, while at nanocomposite concentrations up to 1.66 µg/mL, the cell death value reached 98.7–99.2% in 10 min [131].

Shi et al. in his work developed a dual-nanostructure modified graphene-based nanocomposite by growing a layer of gold nanostructures on the synthesized iron oxide nanoparticles on top of graphine oxide (GO-IONP), obtaining a GO-IONP-Au nanocomposite (graphine oxide-iron oxide nanoparticle and gold nanocomposite), which was then functionalized with PEG. Female BALB/c mice bearing 4T1 tumors were intratumorally injected with 40 mL of 50 mg/mL Pegylated nanocomposite GO-IONP-Au-PEG and then subjected to the 808-nm laser irradiation at the power density of 0.75 W/cm^2^ for 5 min. The study showed that the surface temperature of the tumor injected with GO-IONP-Au-PEG rapidly increased to about 55 °C within 5 min of laser irradiation [132].

An overview of cancer thermal ablation using nanoshells and nanocomposites is presented in Table 6.

## 8. Conclusions

Despite a wide range of research conducted for the thermal therapy of cancer tumor, there are still existing limitations. It is hard to obtain a highly accurate method for temperature monitoring within the whole tumor due to the inhomogeneous distribution of MNPs. A key factor in the success of any nanoparticle-based therapy is the ability to understand and/or control nanoparticle accumulation within the tumor. Thus far, scientists have found many ways to destroy cancer cells in vitro and in vivo in mice, but the translation to commercial clinical use has not yet occurred. Nanomaterials offer a wide range of magnetic and optical properties that can be implemented in cancer therapy under different ablation sources (Table 7). All the studies described in this paper used different nanomaterials, but factors such as size, shape, surface coating, injected dose, animal model, and cancer model were all different, and can make a comparison between the studies a significant challenge. The importance of size on thermal therapy is well investigated and revealed that it is beneficial to use the particles that were between 10–40 nm. However, larger nanoparticles can also be administered into the tumor, but there are still some gaps in the research regarding the heterogeneity of endothelial cells gaps and the penetration of nanoparticles. Also, despite the demonstrations of scaled-up synthesis, nanomaterial synthesis is notorious for being irreproducible, and no two nanoparticle batches are the exact same. Variation in nanoparticle structure/composition may affect tumor-targeting ability and penetration along with photothermal heating efficiency. In addition, the toxicity of nanomaterials is not well understood, and could also vary with structure/composition [34]. Among the disadvantages and key challenges to employing thermal therapy using nanomaterials are the inhomogeneous distribution of nanoparticles, lack of noninvasive technique for biodistribution assessment, lack of treatment planning tools to predict temperature distributions, and increasing patient’s complexity due to the injection of nanoparticles. Therefore, further work and broad investigations are required in this field. 

## Figures and Tables

**Table 1:** Summary of MNPs application in thermal ablation of cancer.

Type of MNP/MNP with a Surface Coating	Nanoparticle Size	Injection Dose/Nanoparticle Concentration	Injection Route	Exposure Conditions	Thermal Ablation Type	Type of Tumor	Temperature, °C	Cell Death	Reference
Fe_3_O_4_	20–100 nm	5 mg of Fe_2_O_3_ per gram of tissue	intratumoral	200 to 240 oersteds, 3 min	RF ablation	dog’s lymph nodes	max 50 °C	N/A	[45]
Fe_3_O_4_	50–100 nm	5 mg of Fe_2_O_3_ per gram of tissue	intratumoral	55,000 cycles/second, 500 oesterds, 30 min	RF ablation	lymph node metastases by	max 50 °C	N/A	[46]
Fe_3_O_4_	10–20 nm	21 mg ± 9 of magnetite per 299 mm^3^ of tumor tissue	intratumoral	1.2–6.5 kA/m; 400 kHz, 242 s	AMF	human breast tissue	max 79 °C	N/A	[53]
Fe_3_O_4_	10 nm	5 ± 0.3 mg magnetite per 100 mg of tumor tissue	intratumoral	400 kHz, 6.5 kA/m, 4 min	AMF	human breast adenocarcinoma cells	max 73 °C at tumor center and 12 °C at tumor periphery	N/A	[20]
Fe_3_O_4_—dextran coated	10–20 nm	107 pg/cells	intratumoral	410 kHz, 10 kA/m, 242 s	AMF	human breast adenocarcinoma cells	max 71 °C at tumor center	N/A	[54]
Fe_3_O_4_—starch coated	11.4 nm ± 0.38	0.32 mg Fe mL^−1^ culture medium	intratumoral	400 kHz, 24.6 kA m^−1^	AMF	breast carcinoma cell line BT474	to 28.2 ± 0.4 °C	N/A	[51]
Fe_3_O_4_—amino-silane coated	15 nm	N/A	intratumoral	100 kHz, 0–18 kA/m	AMF	RG-2 glioma cells	max 43–47 °C	N/A	[55]
Fe_3_O_4_	20 nm	11.4 mL per 100 mg of tumor tissue	interstitial	100 kHz, 2.5–15 kA/m	AMF	prostate cancer	max 55 °C	N/A	[57]
Fe_3_O_4_—amino-silane coated	15 nm	112 mg/mL	transperineally	100 kHz, 2.5–18.0 kA/m, 60 min	AMF	prostate cancer	max 50 °C	N/A	[56]
Fe_3_O_4_—amino-silane coated	15 nm	200–400 µl of MNP per 0.5 mL/cm^3^ tumor volume and 120 mg/mL	intratumoral	100 kHz, 18.0 kA/m	AMF	prostate cancer	max 54.88 °C centrally and 41.28 °C—peripherally	N/A	[58]

**Table 2:** Summary of AuNPs application in the thermal ablation of cancer (GFR: epidermal growth factor receptor).

Type of NP/NP with a Surface Coating	Nanoparticle Size	Injection Dose/Nanoparticle Concentration	Injection Route	Exposure Conditions	Thermal Ablation Type	Type of Tumor	Temperature, °C	Cell Death	Reference
AuNP—anti-EGFR antibody coated	40 nm	N/A	intratumoral	57 W/cm^2^, 514 nm, 4 min	Laser photothermal therapy	Epithelial carcinoma HaCaT cells	N/A	100%	[72]
AuNP—citrate coated	13 nm	N/A	intratumoral	10–100 W, 7 min	Radiowave ablation	HepG2 cancerous cells	>50 °C	80%	[73]
AuNP	5 nm	67 µM/L	intratumoral	13.56 MHz, 5 min	RF ablation	Hepatocellular (Hep3B) and Pancreatic cancerous cells (Panc-1)	N/A	99.8 ± 3.1 Hep3B 96.5 ± 8.4 Panc-1	[74]
AuNP—anti-EGFR coated	N/A	N/A	intratumoral	200 nW	Laser photothermal therapy	Cancerous cell	70–80 °C	N/A	[69]
AuNP—anti-EGFR coated	20 nm	N/A	intratumoral	13.56 MHz, 200 S, 10−15 kV/m	RF ablation	Pancreatic cancerous cell line	N/A	Panc-1 61%	[76]
AuNP—PAM4 hemi-antibody coated and AuNP—C225 antibody-coated	36.9 ± 1.5 nm 32.6 ± 0.7 nm	100 µg/mL	*invivo*	600 W, 10 min	RF ablation	Panc-1 and Capan-1 pancreatic carcinoma cell lines	N/A	N/A	[75]

**Table 3:** Summary of Cu-based nanoparticles application in the thermal ablation of cancer (CuSNPs: hollow copper sulfide nanoparticles).

Type of Nanoparticle/Nanoparticle with a Surface Coating	Nanoparticle Size	Injection Dose/Nanoparticle Concentration	Injection Route	Exposure Conditions	Thermal Ablation Type	Type of Tumor	Cell Death	Temperature, °C	Reference
Thioglycolic acid-stabilized CuS NPs	3 nm	4.6 g/cm^3^, 770 µM	intratumoral	24 W/cm^2^ for 5 min	photothermal ablation	HeLa cells	55.6 ± 5.8%	Increased to 12.7 °C	[79]
Chitosan-coated HCuSNPs	10 × 12 nm		intratumoral		photothermal ablation				[81]
Phospholipid-PEG coated– CuS NPs	3.8 nm	400 mg/mL^−1^	intratumoral	1.0 W cm^2^, 8 min	Photothermal ablation	HeLa cells	>80%	Max 59.2 °C	[82]
PEG coated—CuS NP	N/A	400 mg/mL, 0.1 mm	intratumoral	2.5 W/cm^2^	Photothermal ablation	Anaplastic thyroid carcinoma	N/A	Max 98 °C	[83]

**Table 4:** Summary of nanorods application in the thermal ablation of cancer (PEG: polyethylene glycol).

Type of Nanorods/Nanorods with a Surface Coating	Nanorod Size/Concentration	Injection Dose/Nanorods Concentration	Injection Rote	Exposure Conditions	Thermal Ablation Type	Type of Tumor	Cell Death	Temperature, °C	Reference
AuNP	5 nm	0.001% volume fraction	intratumoral	1.25 W/cm^2^, 300 s	photothermal therapy	Skin tumor	Total cell death	75 °C at tumor surface, 43–48 °C at tumor depth	[93]
PEGylated gold nanorods– C225 antibody	3 mm	4 mg/mL	intratumoral	20 W/cm^2^ to 90 W/cm^2^, from 30 s to 3 min	photothermal laser ablation	HTB-9 cells	N/A	N/A	[94]
PEGylated gold nanorods	dimensions 12 nm in width and 50 nm in length	N/A	intravenous	3 min	photothermal therapy	HSC-3 human squamous carcinoma cells	>90%	N/A	[97]
PEG-coated gold nanorods	N/A	N/A	intratumoral	2 W cm^–2^, 5 min.	photothermal ablation	Cancer tumor	N/A	50–52 °C	[98]
gold nanorods	N/A	N/A	intratumoral	30 J/cm^2^, 30 s	photothermal laser ablation	human KB cells	N/A	Increased by 5 °C	[86]
PEG-coated gold nanorods	N/A	20 mg Au/kg in PBS	intravenous	2 W cm^–2^, 5 min	photothermal ablation	MDA-MB-435 human cancerouscells	Within 10 days all the irradiated, PEG-NR-targeted tumors completely disappeared	over 70 °C	[91]
gold nanorods	N/A	N/A	intratumoral	20 W/cm^2^, 4 to 20 min	photothermal ablation	prostate cancerouscells	N/A	N/A	[92]
gold nanorods	N/A	N/A	intratumoral	1.4 and 2 W/cm^2^, and 0.5, 1, 2, and 5 min	photothermal ablation	MDA-MB-231 human breast cancerous cells	N/A	About 55 °C	[95,96]

**Table 5:** Summary of carbon nanotubes application in the thermal ablation of cancer.

Type of Carbon Nanotubes/Carbon Nanotubes with a Surface Coating	Carbon Nanotube Size	Injection Dose/Carbon Nanotube Concentration	Injection Route	Exposure Conditions	Thermal Ablation Type	Type of Tumor	Cell Death	Temperature, °C	Reference
SWNT–polymer coated	N/A	50 mg/mL	intratumoral	600 W, 13.56 MHz	RF ablation	human cancer cell lines (HepG2, Hep3B and Panc-1	100%	Increase by 1.6 °C per second	[114]
Carbon nanotube—anti-Her2+ antibody coated	N/A	N/A	intratumoral	9.5 W cm^−2^, 4 min	laser ablation	Her2+ human breast carcinoma cancer cells	90%	N/A	[115]
Carbon nanotube—(KFKA)_7_ –peptide coated	N/A	0.75 μg/mL for colon26 cells 2.5 μg/mL for HepG2 cells	intratumoral	30 s	photothermal therapy	Colon and HepG2 cells	N/A	43 °C	[116]
Multi-walled carbon nanotube	900 nm	N/A	intratumoral	15.3 W/cm^2^, 5 min	laser ablation	prostate cancer cell line (PC3) and murine renal cancer cell line (RENCA)	N/A	43 °C	[117]
Carbon nanotube— human albumin protein coated	N/A	N/A	Ex vivo	5 W/cm^2^, 20 min	laser-mediated ablation	pancreatic cancer Panc-1 cells	N/A	29.3 °C at tumor centre	[118]

**Table 6:** Summary of gold nanoshells/nanocomposite application in thermal ablation of cancer (NIR: near infrared).

Type of Nanoshells/Nanocomposites (Core/Shell)	Size and/or Core/Shell Thickness	Injection Dose/Concertation	Injection Route	Exposure Conditions	Thermal Ablation Type	Type of Tumor	Temperature, °C	Cell Death	Reference
Gold nanoshells (silica/gold)	110 nm/10 nm	N/A	intratumoral	35 W/cm^2^, 7 min	photothermal therapy	breast cancer SK-BR-3 cells	>37.4 °C	N/A	[119]
Gold nanoshells (gold/PEG)	110 nm/8–10 nm	100 µL of 2.4 × 10^11^ nanoshells/mL solution	intratumoral	10 days	laser ablation	CT26.WT murine colon carcinoma tumor cells	50 °C	100%	[121]
Gold nanoshells (silica/gold)	110 ± 11 nm/10 nm	8.5 µL/gm body weight	intratumoral	21 days	NIR laser ablation	prostate cancer tumor	65.4 °C	93%	[126]
anti-HER2—silica core nanoshell anti-IL13Ra2 antibody—silica core nanoshell (silica/gold)	100 nm and 10 nm	32.61 and 48.52 mg/g	intratumoral	N/A	photothermal ablation	medulloblastoma and glioma cell lines	N/A	100%	[126]
Gold nanoshells (silica/gold)	150 nm	N/A	intratumoral	3.5 W for 3 min	laser ablation	canine prostate cancer	N/A	100%	[128]
Graphitic carbon coated C–Co-NPs	7 nm	20 μg mL^−1^	intratumoral	350 kHz, 5 kW, 10 min	RF ablation	HeLa cells	N/A	98%	[129]
Fe_3_O_4_ nanoparticles– silica shell (silica/Fe_3_O_4_)	N/A	1.66 µg/mL	intratumoral	350 kHz, 10 min	RF ablation	Panc-1 cell line	N/A	98.7– 99.2%	[131]
GO-IONP-Au-PEG	N/A	50 mg/mL	intratumoral	0.75 W/cm^2^, 5 min	Laser ablation	4T1 tumor cells	Max 55 °C	N/A	[132]

**Table 7:** Summary on types of nanoparticles and ablation techniques.

Type of Nanoparticles/Source of Ablation	RF	MW	Laser Ablation	Photothermal Ablation
Magnetic nanoparticle				
Gold nanoparticle				
Cu-based nanoparticle				
Nanorod				
Carbon nanotubes				
Nanoshell/Nanocomposite				
